# Microvesicles derived from human Wharton’s jelly mesenchymal stem cells enhance autophagy and ameliorate acute lung injury via delivery of miR-100

**DOI:** 10.1186/s13287-020-01617-7

**Published:** 2020-03-13

**Authors:** Wen-xia Chen, Jun Zhou, Sha-sha Zhou, Yu-dan Zhang, Tong-yu Ji, Xiao-li Zhang, Shu-min Wang, Tao Du, De-gang Ding

**Affiliations:** 1 Department of Pediatrics, The First Affiliated Hospital of Henan University of Traditional Chinese Medicine, Zhengzhou, 450000 Henan China; 2 Department of Urology, Henan University People’s Hospital; Henan Provincial People’s Hospital, Zhengzhou, Henan 450003 China; 3 Department of Urology, Henan Provincial People’s Hospital, Zhengzhou University People’s Hospital, Zhengzhou, 450003 Henan China

**Keywords:** Microvesicles, Mesenchymal stem cells, Acute lung injury, Autophagy, miR-100

## Abstract

**Objectives:**

Microvesicles (MVs) derived from human Wharton’s jelly mesenchymal stem cells (MSC-MVs) were demonstrated to ameliorate acute lung injury (ALI). We have previously found that MSC-MV-transferred hepatocyte growth factor was partly involved in their therapeutic effects. Since MSC-MVs also contained a substantial quantity of miR-100, which plays an important role in lung cancer and injury, we speculated that miR-100 might similarly account for a part of the therapeutic effects of MSC-MVs.

**Methods:**

MSCs were transfected with miR-100 inhibitor to downregulate miR-100 in MSC-MVs. A rat model of ALI and cell injury in rat type II alveolar epithelial cell line (L2) was induced by bleomycin (BLM). A co-culture model of alveolar epithelial cells and MSC-MVs was utilized to examine the therapeutic role of MSC-MVs and mechanism.

**Results:**

MSC-MV treatment attenuated BLM-induced apoptosis and inflammation in BLM-treated L2 cells and ameliorated BLM-induced lung apoptosis, inflammation, and fibrosis in BLM-induced ALI rats. The beneficial effect of MSC-MVs was partly eliminated when miR-100 was knocked down in MSCs. Moreover, MSC-MV-transferred miR-100 mediated the therapeutic effect of MSC-MVs in ALI through enhancing autophagy by targeting mTOR.

**Conclusion:**

MSC-MVs enhance autophagy and ameliorate ALI partially via delivery of miR-100.

## Introduction

Acute lung injury (ALI) is a serious condition characterized by a severe inflammatory response of lung injuries and damage to the microvascular permeability, frequently resulting in death [1]. Despite improvements in supportive care and antibiotic use, the morbidity and mortality of ALI remain high.

In recent years, abundant animal studies provided strong evidence that administration of mesenchymal stem cells (MSCs) could effectively improve recovery from ALI [2, 3]. Increasing evidence indicates that MSCs exert therapeutic effects, at least partially, by microvesicles (MVs) released from MSCs (MSC-MVs) [4, 5]. MVs are nuclear plasma membrane-bound cell fragments, 50 to 200 nm in size, released from the endosomal compartment as exosomes or shed from the surface membranes of most cell types [6]. MVs play a crucial role in cell-to-cell communication, and MSC-MVs have been shown to attenuate ALI by transferring various bioactive molecules including proteins/peptides, mRNAs, microRNAs, lipids, and organelles with anti-apoptotic, anti-oxidative, pro-regenerative, pro-metabolomic, and immunoregulatory properties to the injured tissue [5–7]. However, the mechanism underlying the therapeutic benefit of MSC-MVs for ALI is not fully illuminated.

The mechanistic target of rapamycin (mTOR) signaling can negatively regulate autophagy, which is a homeostatic, catabolic degradation process to sustain cellular metabolism [8–10]. Studies have shown that the mTOR signaling and its regulated autophagy play an important role in various diseases including ALI [1, 11]. Hu et al. [12] demonstrated that activation of mTOR in the epithelium promoted lipopolysaccharide (LPS)-induced ALI, likely through downregulation of autophagy and the subsequent activation of NF-κB signaling pathway, indicating that inhibition of mTOR in pulmonary epithelial cells may represent a promising therapeutic strategy for preventing ALI.

Studies have shown that miRNAs are involved in the pathogenesis of lung injury [13, 14]. Our bioinformatics analysis (TargetScan) revealed that mTOR, the negative regulator of autophagy, was a putative target of some miRNAs. Among them, miR-100 has been shown to confer protection in lung injury. Liu et al. reported that inhibition of miR-100 aggravated the LPS-induced cell injury in LPS-treated WI-38 human lung fibroblasts, a cell model of pulmonary injury [15]. Considering the important role of mTOR in promoting ALI, we speculated that miR-100 might confer protection in lung injury by positively regulating autophagy via targeted suppressing mTOR, which has not been reported yet and needs to be defined. Through miRNA microarray, Zhao et al. [16] found that miR-100 was expressed in MSC-MVs and significantly upregulated in MSC-MVs treated with interferon-γ (IFN-γ), which is essential to induce the immunosuppressive effects of MSCs [17]. Therefore, it is suggested that miR-100 may be involved in the immunosuppressive regulation of MSCs and MSC-MVs. Combined with the abovementioned therapeutic effect of MSC-MVs and mTOR inhibition for ALI, we hypothesized that MSC-MVs might transfer miR-100 into injured epithelial cells where miR-100 could enhance autophagy by targeting and inhibiting mTOR expression, thereby ameliorating ALI.

## Materials and methods

### Isolation and identification of hWJMSC-MVs

This experiment was approved by the Ethics Committee of the First Affiliated Hospital of Henan University of Traditional Chinese Medicine. Fresh human umbilical cords were collected from newborn infants with the written consent of their parents. The human Wharton’s jelly MSCs (hWJMSCs) were isolated from the mesenchymal tissues as described in our previous study [4]. The hWJMSCs at the third passage were collected and prepared for isolating MVs. The obtained MVs were authenticated by transmission electron microscope (TEM) and flow cytometry (FCM) [4].

### Cell culture

Rat type II alveolar epithelial cell line L2 was purchased from American Type Culture Collection (ATCC, Manassas, VA, USA) and cultured in RPMI 1640 medium (Life Technologies) containing 10% fetal bovine serum (FBS, Gibco, Thermo Fisher Scientific, Waltham, MA, USA). The medium was changed every 48 h.

### miRNA mimic and miRNA inhibitor transfection

miR-100 inhibitor (miR-100I), inhibitor negative control (NCI), miR-100 mimic, and mimic NC were purchased from Shanghai GenePharma (Shanghai, China). MSCs at 80% confluence were transfected with miR-100I or NCI. L2 cells at 80% confluence were transfected with miR-100 mimic or mimic NC. Cell transfection was performed using Lipofectamine™ 2000 (Invitrogen; Thermo Fisher Scientific) in 12-well plates at a dose of 50 nM. Following transfection for 48 h, cells were harvested for determination of miR-100 expression.

### Cell treatment

To assess the effect of MSC-MV-mediated delivery of miR-100 on bleomycin (BLM)-induced ALI, L2 cells were randomly divided into 5 subgroups: control, BLM, BLM+MVs, BLM+NCI-MVs, and BLM+miR-100I-MVs. BLM was used at the concentration of 1 mg/L.

To examine the effect of miR-100 on BLM-induced ALI, L2 cells were divided into 4 subgroups: control, BLM, BLM+mimic NC, and BLM+miR-100 mimic.

To further determine whether miR-100 modulated lung injury via regulating autophagy, L2 cells were randomized into 4 subgroups, namely, BLM, BLM+MVs, BLM+MVs+DMSO, and BLM+MVs+MHY1485.

### MV labeling and uptake

Purified MSC-MVs were labeled with 1 μM Dil (Invitrogen) as previously described [18]. Briefly, MSC-MVs were incubated with 1 μM Dil for 5 min with gentle agitation. Excess dye from the labeled MVs was removed by ultracentrifugation at 100,000*g* for 1 h at 4 °C. The final pellets were resuspended in PBS. Subsequently, Dil-labeled MVs were co-cultured with L2 cells for 6 h, and then, L2 cells were fixed in 4% paraformaldehyde and stained with Hoechst 33342. The distribution and intensity of fluorescence were observed by fluorescence microscopy to analyze MV uptake by L2 cells.

### Detection of apoptosis

An annexin V-fluorescein isothiocyanate (FITC)/propidium iodide (PI) cell apoptosis kit (Invitrogen) was used to quantify L2 cell apoptosis according to the instructions. Briefly, L2 cells were washed twice with PBS and stained with annexin V-FITC and PI at 4 °C in the dark for 30 min. Flow cytometry was performed, and the statistical diagrams were drawn by FlowJo 7.6 software (Treestar, Inc., Ashland, OR, USA).

### Dual-luciferase reporter assay

Briefly, 3′-untranslational region (3′-UTR) of mTOR was amplified by PCR and then cloned into pGL3 vector. For luciferase assay, L2 cells were seeded in 24-well plates. When it reached 70 to 80% confluence, cells were co-transfected with wild type (WT) or mutant (Mut) mTOR 3′-UTR luciferase reporter plasmids, together with mimic NC or miR-100 mimic by Lipofectamine 2000™ (Invitrogen). A Dual-Luciferase Reporter Assay Kit (Promega Corporation, Madison, WI, USA) was applied to analyze luciferase activity at 24 h post-transfection.

### Animals and experimental groups

All the animal experimental protocols were in accordance with national animal experiment requirements that were approved by the Ethics Committee of the First Affiliated Hospital of Henan University of Traditional Chinese Medicine. Specific pathogen-free (SPF) Sprague-Dawley rats (male, weighing 180–200 g) were given free access to food and water at controlled condition (temperature, 22 ± 2 °C; humidity, 55 ± 5%), with 12 h light/dark cycle. After 1 week of acclimatization, rats were randomly divided into the following 5 groups (*n* = 10/group):

1. Control: Rats were intratracheally administrated with equal volume of normal saline instead of BLM.
2. ALI+vehicle: Rats were intratracheally administrated with BLM (4 mg/kg) and PBS as MV vehicle control.
3. ALI+MVs: Rats were intratracheally administrated with MSC-MVs (10 μl containing ~ 1 × 10^6^) 2 days after BLM (4 mg/kg) treatment.
4. ALI+NCI-MVs: Rats were intratracheally administrated with MVs (10 μl containing ~ 1 × 10^6^) derived from NCI-transfected MSCs 2 days after BLM (4 mg/kg) treatment.
5. ALI+miR-100I-MVs: Rats were intratracheally administrated with MVs (10 μl containing ~ 1 × 10^6^) derived from miR-100I-transfected MSCs 2 days after BLM (4 mg/kg) treatment.

After 48 h or 1 week, rats were euthanatized by inhalation of ether. The bronchoalveolar lavage fluid (BALF) samples and lung tissues were collected for the following experiments.

### Measurement of total protein content in the BALF

Total protein concentration in BALF was measured to examine pulmonary permeability. Briefly, bronchoalveolar lavage (BAL) was performed by instilling 1 ml of 0.9% saline through the tracheal cannula, and BALF was centrifuged at 10,000*g* for 5 min. Supernatants were collected and stored at − 80 °C until assayed. BALF was mixed with Bio-Rad solution (Bio-Rad Laboratories, Richmond, CA, USA) for 10 min of incubation at room temperature. The absorbance of the fluid was then read at 595 nm on a spectrophotometer. The protein concentration was determined by comparison with the standard curve using bovine serum albumin.

### Cell counts

For determination of total cell numbers in the BALF, BALF samples were centrifuged at 1500*g* for 10 min; the cell pellet was resuspended in 50 μl PBS and counted with a hematology analyzer (Siemens, Germany). For neutrophil quantification, the cell pellets following centrifugation from BALF samples were diluted in PBS, fixed in methanol, and stained with Wright stain. Neutrophil quantification was achieved via light microscopy by two experienced and blinded members.

### TdT-mediated dUTP nick-end labeling staining

Briefly, the lung tissues were fixed in 4% paraformaldehyde, dehydrated with a graded series of ethanol, and then embedded in paraffin before being cut into 5-μm-thick sections. Apoptotic cells in the lung tissues were detected by TdT-mediated dUTP nick-end labeling (TUNEL) staining using the In Situ Cell Death Detection kit (Roche Diagnostics, GmbH, Mannheim, Germany) following the manufacturer’s protocols. The sections were sealed, and images were acquired under a light microscope (Olympus Co., Tokyo, Japan). The number of TUNEL-positive cells (brown) was counted from 5 fields and compared with the total cells using Image-Pro Plus 6.0 software and expressed as a percentage.

### Histological examinations

Briefly, the 5-μm-thick lung sections were stained with the Masson’s Trichrome Stain Kit (Solarbio, Beijing, China) to assess the level of lung fibrosis following the routine staining procedures. The collagen volume fraction (CVF) was calculated as ratio of stained collagen area to total area using Image-Pro Plus 6.0 software. For hematoxylin and eosin (H&E) staining, the 5-μm-thick lung sections were stained with H&E Kit (Sigma-Aldrich, St. Louis, MO, USA) according to the routine staining procedure. Images were analyzed using a light microscope (Olympus Co.). The images were reviewed by two senior pathologists with a double-blind method. The injury was scored with a semi-quantitative grading system based on structural changes, including edema, alveolar and interstitial hemorrhage, and inflammatory cell sequestration [19].

### Enzyme-linked immunosorbent assay

The levels of tumor necrosis factor (TNF)-α (R&D Systems, Minneapolis, MN, USA), interleukin (IL)-6 (R&D Systems), and IL-8 (Nanjing SenBeiJia Biological Technology, Nanjing, China) in cell supernatant or rat BALF were measured using enzyme-linked immunosorbent assay (ELISA) kits following the manufacturer’s protocol.

### Quantitative real-time PCR

Total RNA was extracted from cultured cells, MVs, or lung tissues using the RNApure Total RNAFast Extraction Kit (BioTeke, Beijing, China) and reverse-transcribed to cDNA using a PrimeScript RT Reagent Kit (TaKaRa, Dalian, China) according to the manufacturer’s protocol. The expression levels of miR-100 were determined using the miRNA quantitative real-time PCR (qRT-PCR) kit (GeneCopoeia, Rockville, MD, USA) in Applied Biosystems 7500 PCR system. The primers for miR-100 were synthesized by Sangon Biotechnology (Shanghai, China). Relative expression of miR-100 was calculated by the 2^−ΔΔCt^ method and normalized to U6.

### Western blot

Total protein was extracted from the cells and tissues in RIPA lysis buffer. Briefly, 40 μg of protein was separated by 10% SDS-PAGE gels and electro-transferred onto PVDF membranes (Millipore, Billerica, MA, USA). The membrane was blocked with 5% (w/v) nonfat dry milk for 2 h followed by an overnight incubation at 4 °C with primary antibodies against mTOR (1:1000, Abcam, Cambridge, MA, USA), LC3-I and LC3-II (1:1000, Abcam), Beclin-1 (1:1000, Abcam), and β-actin (1:1000; Santa Cruz Biotechnology, Dallas, TX, USA). Subsequently, membranes were incubated for an additional 2 h with HRP-conjugated secondary antibody (Abcam) at room temperature. Finally, the bands were detected by enhanced chemiluminescence (ECL) kit. β-Actin was used as the loading control.

### Statistical analysis

All statistical analyses were performed using SPSS 21.0 (SPSS, Inc., Chicago, IL, USA). Statistical significance was analyzed using Student’s *t* test between two groups and one-way analysis of variance (ANOVA) among three or more groups. The quantitative statistics were presented as the mean ± standard deviation (SD) from three independent experiments. Significance was set at *p* < 0.05.

## Results

### MSC-MVs attenuate the BLM-induced apoptosis and inflammation via transferring miR-100

**Fig. 1:**
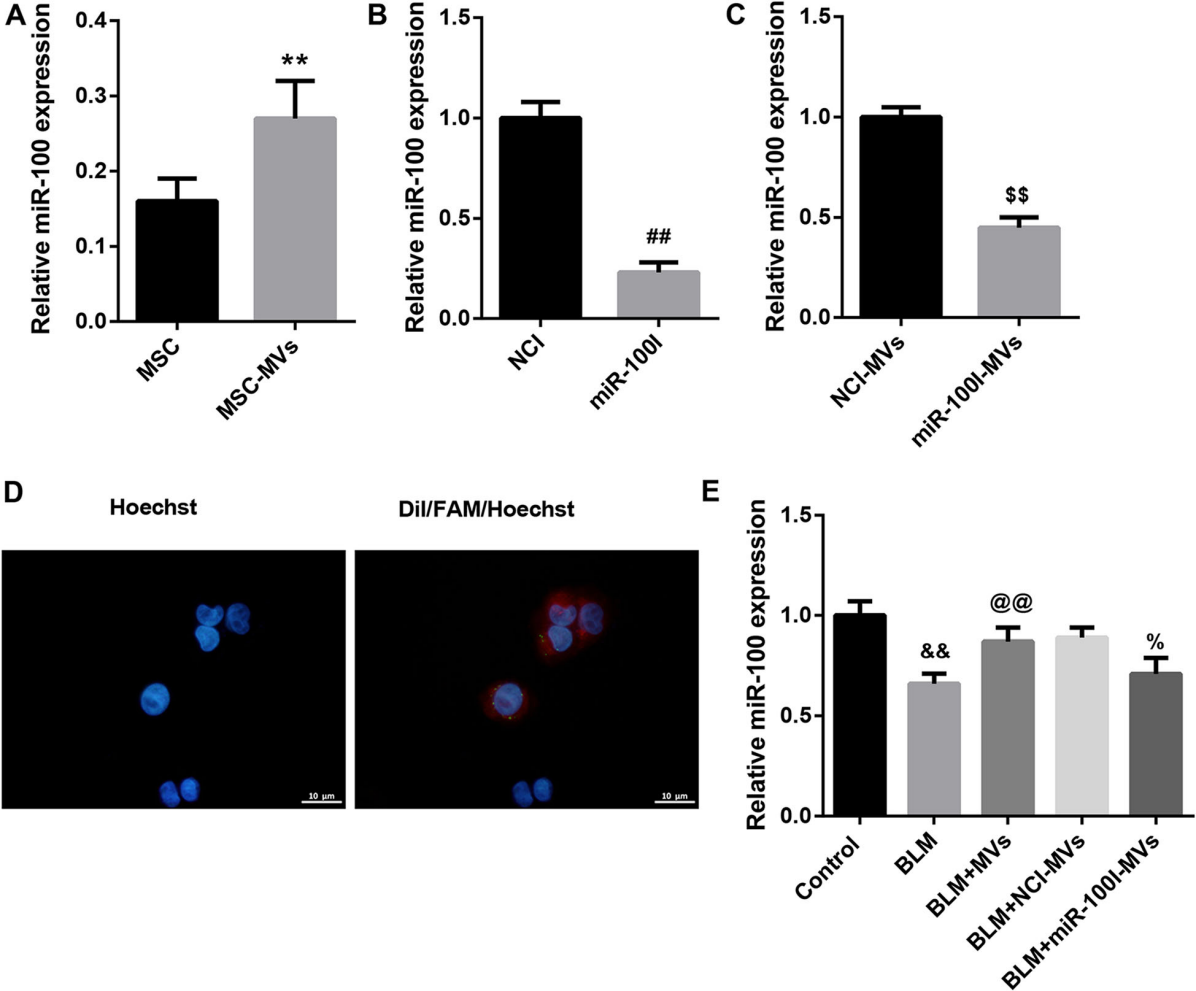
MSC-MVs mediate transfer of miR-100 to BLM-treated L2 cells. **a** qRT-PCR analysis of miR-100 expression in MSCs and MSC-MVs. **b** qRT-PCR analysis of miR-100 expression in NCI-transfected MSCs and miR-100I-transfected MSCs. **c** qRT-PCR analysis of miR-100 expression in MVs derived from NCI-transfected MSCs (NCI-MVs) and MVs from miR-100I-transfected MSCs (miR-100I-MVs). **d** The MVs derived from FAM (green)-miR-100-transfected MSCs were labeled with Dil (red) and co-cultured with rat type II alveolar epithelial cell line L2 (nuclei stained with Hoechst 33342, blue). The distribution and intensity of fluorescence were observed by confocal laser microscopy to analyze MV uptake by L2 cells. **e** qRT-PCR analysis of miR-100 expression in L2 cells in the groups of control, BLM, BLM+MVs, BLM+NCI-MVs, and BLM+miR-100I-MVs. Data are presented as the means ± SD (*n* = 3). **p < 0.01, vs. MSC; ^##^*p* < 0.01, vs. NCI; ^$$^*p* < 0.01, vs. NCI-MVs; ^&&^*p* < 0.01, vs. control; ^@@^*p* < 0.01, vs. BLM; ^%^*p* < 0.05, vs. BLM+NCI-MVs

We first examined miR-100 expression in MSCs and MSC-MVs. qRT-PCR analysis confirmed that both MSCs and MSC-MVs expressed miR-100 (Fig. 1a). To further investigate whether miR-100 was transferred into L2 cells, we knocked down miR-100 in MSCs using a synthesized miR-100 inhibitor (miR-100I). qRT-PCR analysis confirmed that miR-100 level was notably decreased in miR-100I-transfected MSCs compared with cells transfected with inhibitor control (NCI) (Fig. 1b). Furthermore, we observed a significant decreased miR-100 level in MVs secreted by miR-100I-transfected MSCs (miR-100I-MVs) compared with those from cells transfected with NCI (NCI-MVs) (Fig. 1c). Moreover, we transfected MSCs with FAM-miR-100 and then labeled the secreted MVs with Dil. FAM-miR-100 signals were green and Dil was red. As expected, both the red and green signals were detected in the cytoplasm of L2 cells exposed to these Dil-labeled MVs under fluorescence microscopy (Fig. 1d). Additionally, MVs significantly restored the decreased miR-100 level in L2 cells due to BLM treatment, and this effect was compromised when L2 cells were treated with miR-100I-MVs (Fig. 1e). These data indicate that MSC-MVs mediate the transfer of miR-100 to BLM-treated L2 cells.

**Fig. 2:**
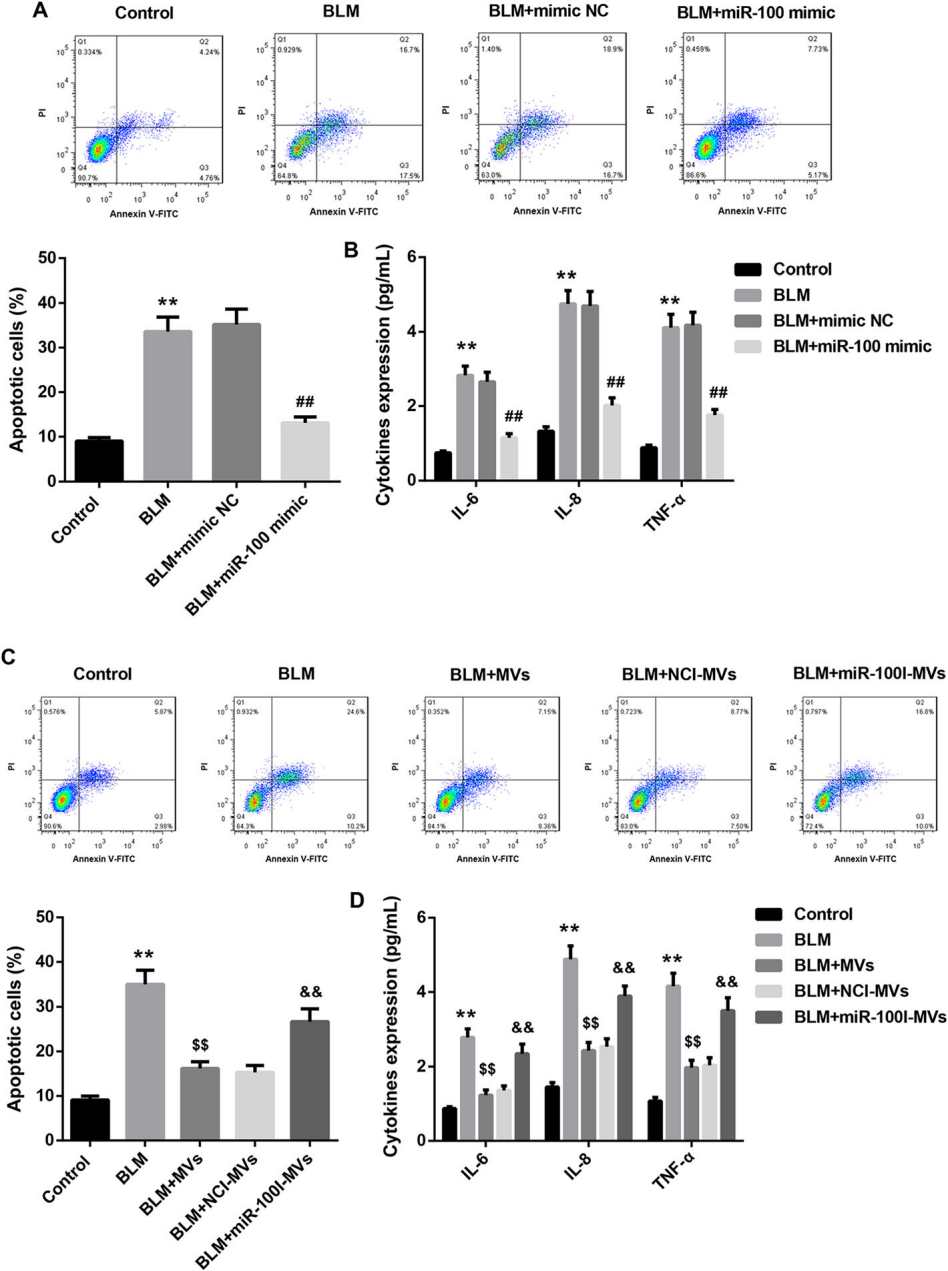
MSC-MVs attenuate the BLM-induced apoptosis and inflammation via transferring miR-100. **a**, **b** Annexin V-FITC/PI apoptotic assay (**a**) and levels of IL-6, IL-8, and TNF-α in cell supernatant (**b**) in L2 cells in the groups of control, BLM, BLM+mimic NC, and BLM+miR-100 mimic. **c**, **d** Annexin V-FITC/PI apoptotic assay (**c**) and levels of IL-6, IL-8, and TNF-α in cell supernatant (**d**) in L2 cells in the groups of control, BLM, BLM+MVs, BLM+NCI-MVs, and BLM+miR-100I-MVs. Data are presented as the means ± SD (*n* = 3). **p < 0.01, vs. control; ^##^*p* < 0.01, vs. BLM+mimic NC; ^$$^*p* < 0.01, vs. BLM; ^&&^*p* < 0.01, vs. BLM+NCI-MVs

To further investigate the effect of MV-transferred miR-100 on BLM-induced ALI, miR-100 mimics were transfected directly into L2 cells. Annexin V-FITC/PI apoptotic assay showed that miR-100 mimic significantly attenuated the BLM-induced cellular apoptosis in L2 cells (Fig. 2a). Furthermore, the levels of pro-inflammatory cytokines (IL-6, IL-8, and TNF-α) in cell supernatant were substantially elevated by BLM treatment, and the pro-inflammatory effect of BLM was effectively attenuated by miR-100 mimic transfection (Fig. 2b). Moreover, for L2 cells treated with MVs, the BLM-induced cellular apoptosis and inflammation were effectively rescued by MV treatment. Importantly, miR-100I-MVs treatment partially attenuated the anti-apoptotic and anti-inflammatory effect of MVs (Fig. 2c, d). Together, these results indicate that the MSC-MVs attenuate the BLM-induced apoptosis and inflammation in L2 cells via transferring miR-100.

### MSC-MV-transferred miR-100 restores the BLM-inhibited autophagy by targeting mTOR

**Fig. 3:**
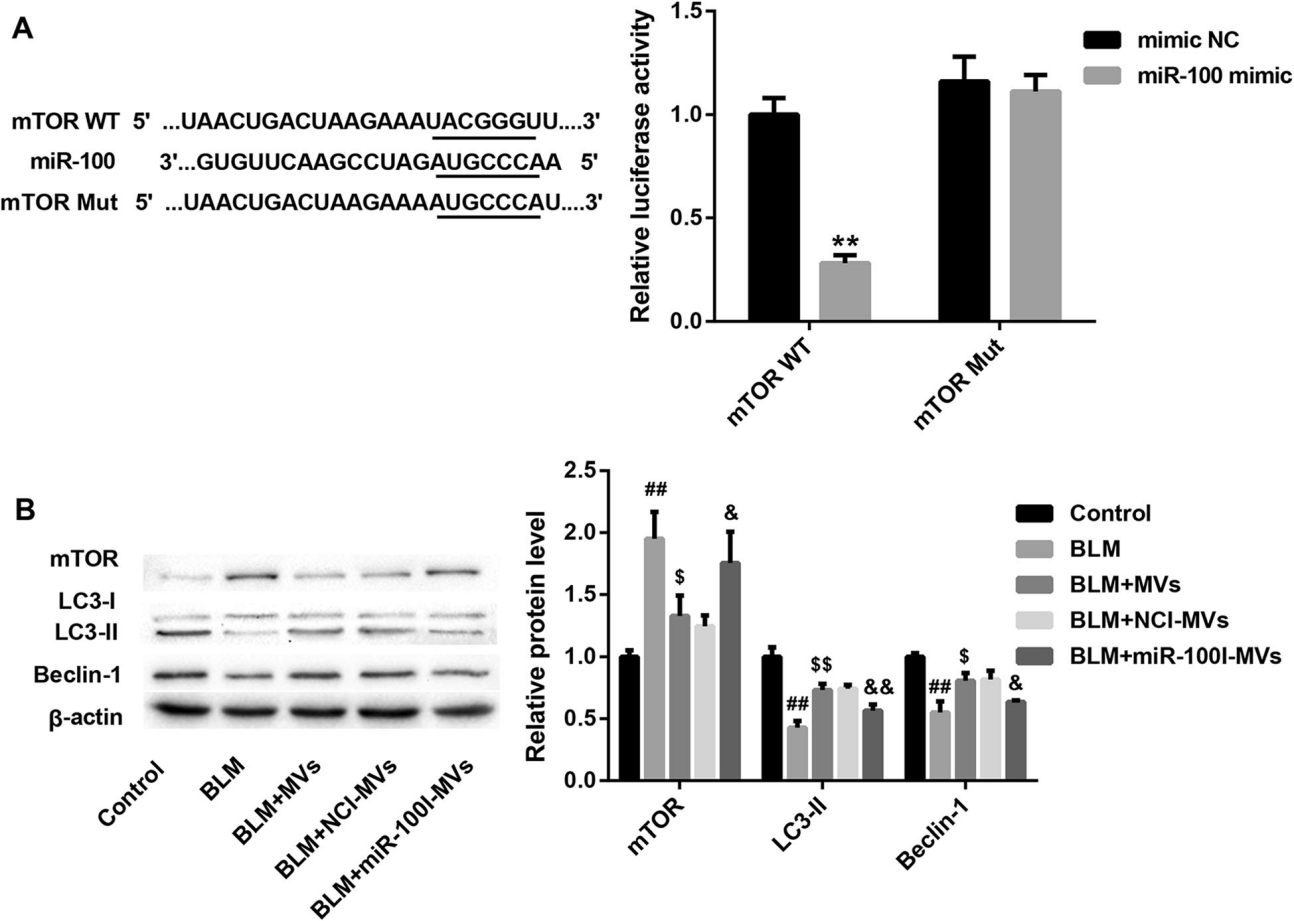
MSC-MVs restore the BLM-inhibited autophagy via transferring miR-100 targeting mTOR. **a** The putative miR-100 binding sites in mTOR (mTOR WT) or and the designed mutant sequence (mTOR Mut) were indicated. Luciferase reporter assay was conducted to evaluate the interaction between mTOR 3′-UTR and miR-100. **b** The protein levels of mTOR and autophagy-related LC3-I, LC3-II, and Beclin-1 in L2 cells in the groups of control, BLM, BLM+MVs, BLM+NCI-MVs, and BLM+miR-100I-MVs. Data are presented as the means ± SD (*n* = 3). **p < 0.01, vs. mimic NC; ^##^*p* < 0.01, vs. control; ^$^*p* < 0.05, ^$$^*p* < 0.01, vs. BLM; ^&^*p* < 0.05, ^&&^*p* < 0.01, vs. BLM+NCI-MVs

Next, we further explored whether the molecular mechanism underlying the anti-apoptotic and anti-inflammatory activity of miR-100 was associated with autophagy regulation by targeting mTOR. Data from luciferase reporter assay showed a decline in luciferase activity of mTOR WT reporter upon introduction of miR-100 mimic. However, miR-100 mimic had no obvious effect on luciferase activity when co-transfected with reporter containing Mut mTOR 3′-UTR (Fig. 3a), suggesting that mTOR was a direct downstream target of miR-100.

We also found that BLM treatment resulted in a distinct upregulation in mTOR protein level, accompanied by a significant downregulation of autophagy-related markers LC3II and Beclin-1 expression in L2 cells, indicating that BLM might suppress autophagy via elevating mTOR level. Of note, MV treatment significantly restored the decreased autophagy level in L2 cells due to BLM treatment, and this effect was compromised when L2 cells were treated with miR-100I-MVs (Fig. 3b). Collectively, these results confirm that MSC-MV-transferred miR-100 restores the BLM-inhibited autophagy by targeting mTOR.

### MSC-MVs attenuate the BLM-induced apoptosis and inflammation via elevating autophagy

**Fig. 4:**
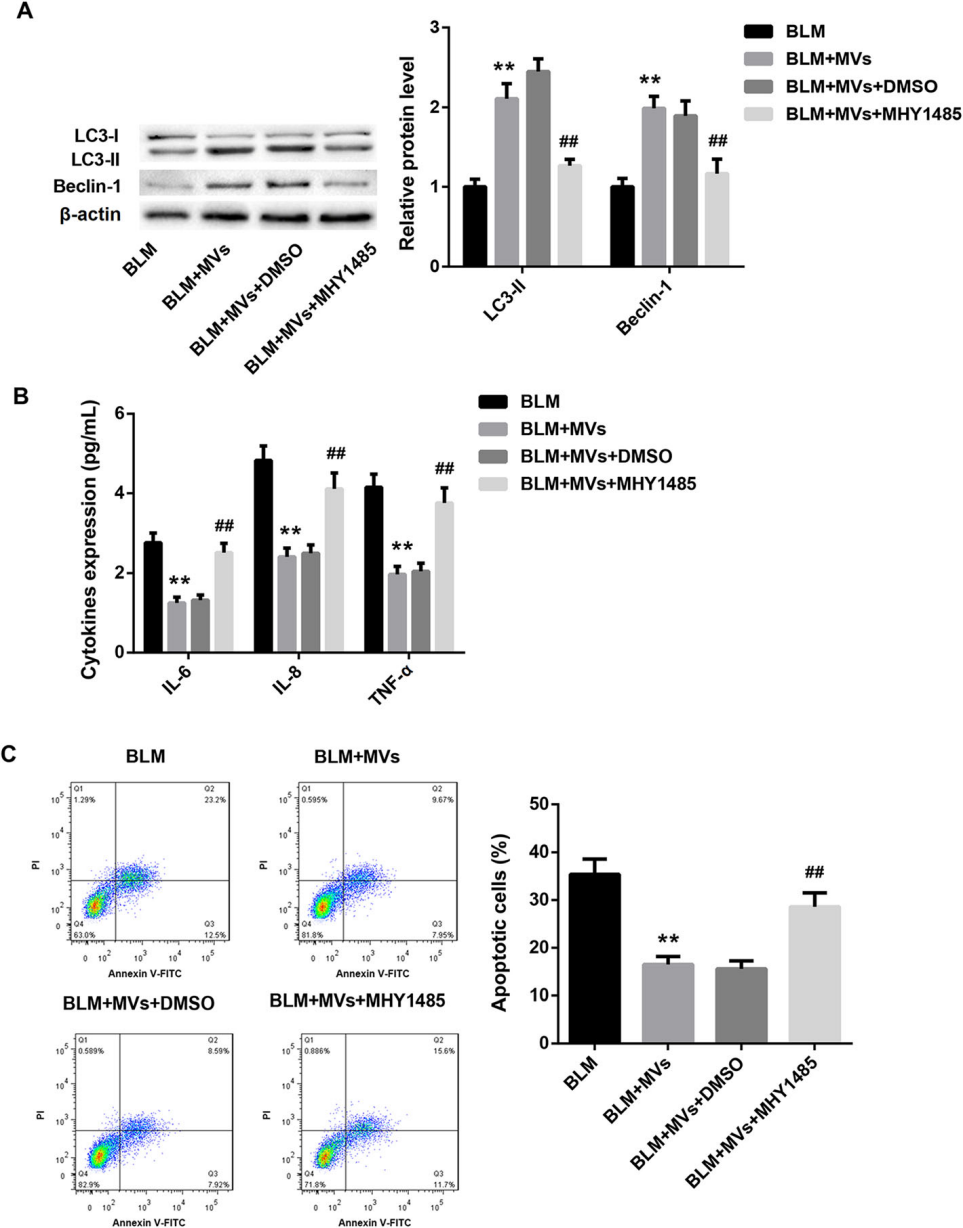
MSC-MVs attenuate the BLM-induced apoptosis and inflammation via elevating autophagy. The protein levels of autophagy-related LC3-I, LC3-II, and Beclin-1 (**a**); levels of IL-6, IL-8, and TNF-α in cell supernatant (**b**); and annexin V-FITC/PI apoptotic assay (**c**) in L2 cells in the groups of BLM, BLM+MVs, BLM+MVs+DMSO, and BLM+MVs+MHY1485. Data are presented as the means ± SD (*n* = 3). **p < 0.01, vs. BLM; ^##^*p* < 0.01, vs. BLM+MVs+DMSO

To further elucidate whether MSC-MVs attenuated the BLM-induced apoptosis and inflammation via elevating autophagy, L2 cells were pre-treated with MHY1485 (a mTOR activator that potently inhibits autophagy) or DMSO (vehicle), followed by treatment with MVs and BLM, both alone and in combination. As expected, MHY1485 pretreatment significantly attenuated the promoting autophagy effect of MVs in BLM-treated L2 cells (Fig. 4a). Importantly, MHY1485 pretreatment effectively attenuated the anti-inflammatory and anti-apoptotic effect of MVs in BLM-treated L2 cells, as manifested by a notable increase in levels of pro-inflammatory cytokines (IL-6, IL-8, and TNF-α) (Fig. 4b) and the percentage of apoptotic cells (Fig. 4c) in the MHY1485 treatment group. These data suggest that MSC-MVs attenuate the BLM-induced apoptosis and inflammation via elevating autophagy.

### MSC-MVs ameliorate BLM-induced ALI via transferring miR-100 in vivo

**Fig. 5:**
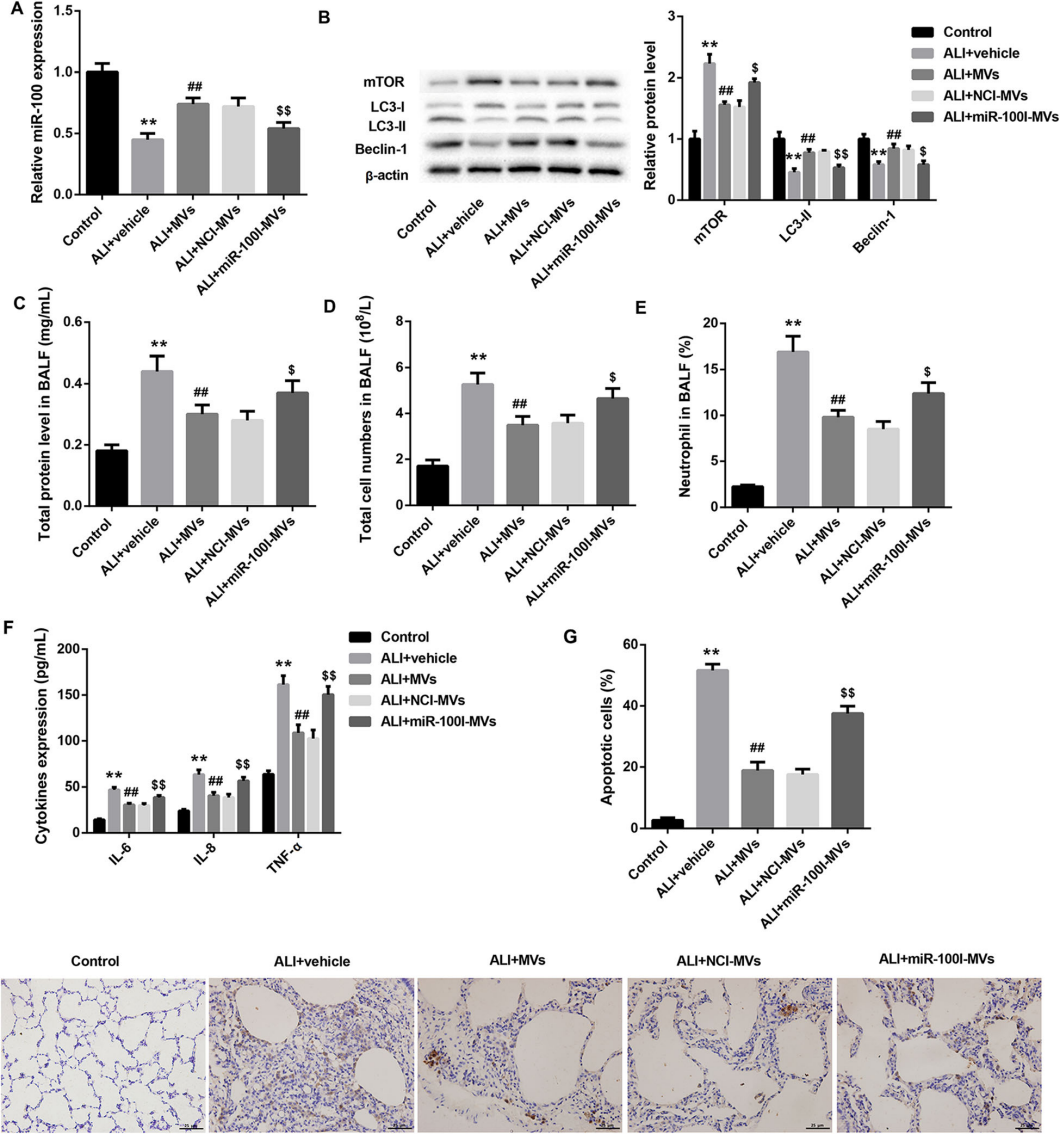
Changes of miR-100 expression, autophagy level, lung apoptosis, and inflammation in ALI rats 48 h following administration with miR-100I-MVs. qRT-PCR analysis of miR-100 in lung tissues (**a**); western blot analysis of protein levels of mTOR, LC3-I, LC3-II, and Beclin-1 in lung tissues (**b**); total protein level in BALF (**c**); total cell numbers in BALF (**d**); neutrophil counts in BALF (**e**); levels of IL-6, IL-8, and TNF-α in BALF determined by ELISA (**f**); and TUNEL staining of apoptotic lung tissue cells (**g**) from ALI rats following 48 h treatment of vehicle (PBS), MVs, NCI-MVs, and miR-100I-MVs. *N* = 10 in each group. **p < 0.01, vs. control; ^##^*p* < 0.01, vs. ALI+vehicle; ^$^*p* < 0.05, ^$$^*p* < 0.01, vs. ALI+NCI-MVs

**Fig. 6:**
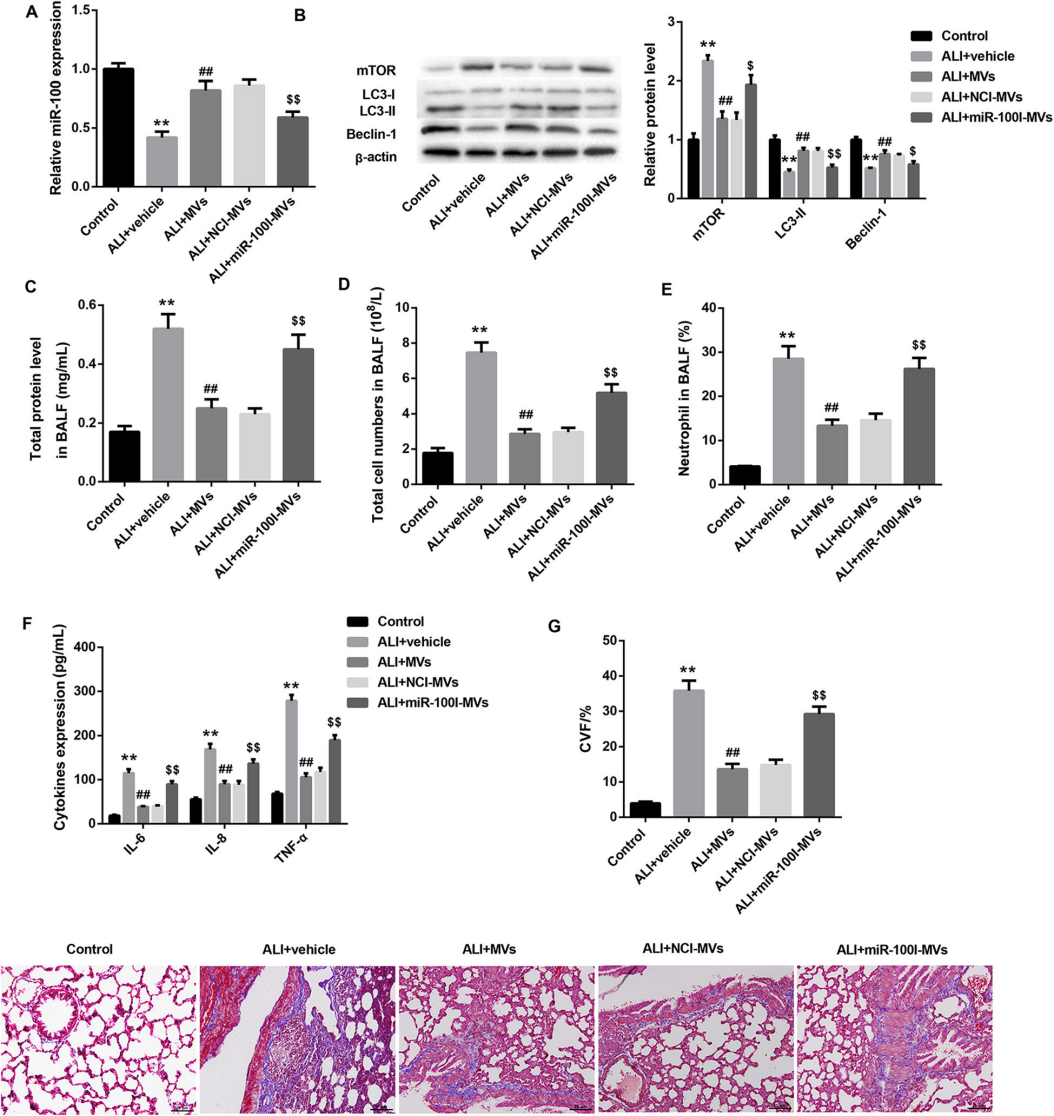
Changes of miR-100 expression, autophagy level, lung fibrosis, and inflammation in ALI rats 1 week following administration with miR-100I-MVs. qRT-PCR analysis of miR-100 in lung tissues (**a**); western blot analysis of protein levels of mTOR, LC3-I, LC3-II, and Beclin-1 in lung tissues (**b**); total protein level in BALF (**c**); total cell numbers in BALF (**d**); neutrophil counts in BALF (**e**); levels of IL-6, IL-8, and TNF-α in BALF determined by ELISA (**f**), and lung fibrosis indicated by Masson’s trichrome staining of lung tissues (**g**) from ALI rats following 1 week treatment of vehicle (PBS), MVs, NCI-MVs, and miR-100I-MVs. *N* = 10 in each group. **p < 0.01, vs. control; ^##^*p* < 0.01, vs. ALI+vehicle; ^$^*p* < 0.05, ^$$^*p* < 0.01, vs. ALI+NCI-MVs

To confirm the in vivo role of MSC-MVs and miR-100 in ALI, we established a rat model of ALI by intratracheal instillation of BLM and treated these ALI rats with vehicle, MVs, NCI-MVs, and miR-100I-MVs. As expected, administration of MSC-MVs for 48 h or 1 week elevated the decreased miR-100 level in rat lungs due to BLM instillation. However, administration of miR-100I-MVs inhibited miR-100 level in rat lungs when compared with the NCI-MV group (Fig. 5a; Fig. 6a).

Furthermore, BLM-challenged rats exhibited markedly increased mTOR protein level, accompanied by significantly decreased levels of LC3 II and Beclin-1 in rat lungs, which was reversed by MSC-MV treatment for 48 h and 1 week. The effect of MSC-MVs on levels of these autophagy-related proteins was effectively abrogated by miR-100I-MVs (Fig. 5b; Fig. 6b). We also found that the total protein content, total cell numbers, neutrophil counts, and levels of pro-inflammatory cytokines (IL-6, IL-8, and TNF-α) in BALF were significantly elevated following BLM administration, which were reduced after 48 h or 1-week treatment of MSC-MVs. However, administration with miR-100I-MSC-MVs for 48 h or 1 week overturned the effect of MSC-MVs (Fig. 5c–f; Fig. 6c–f).

**Fig. 7:**
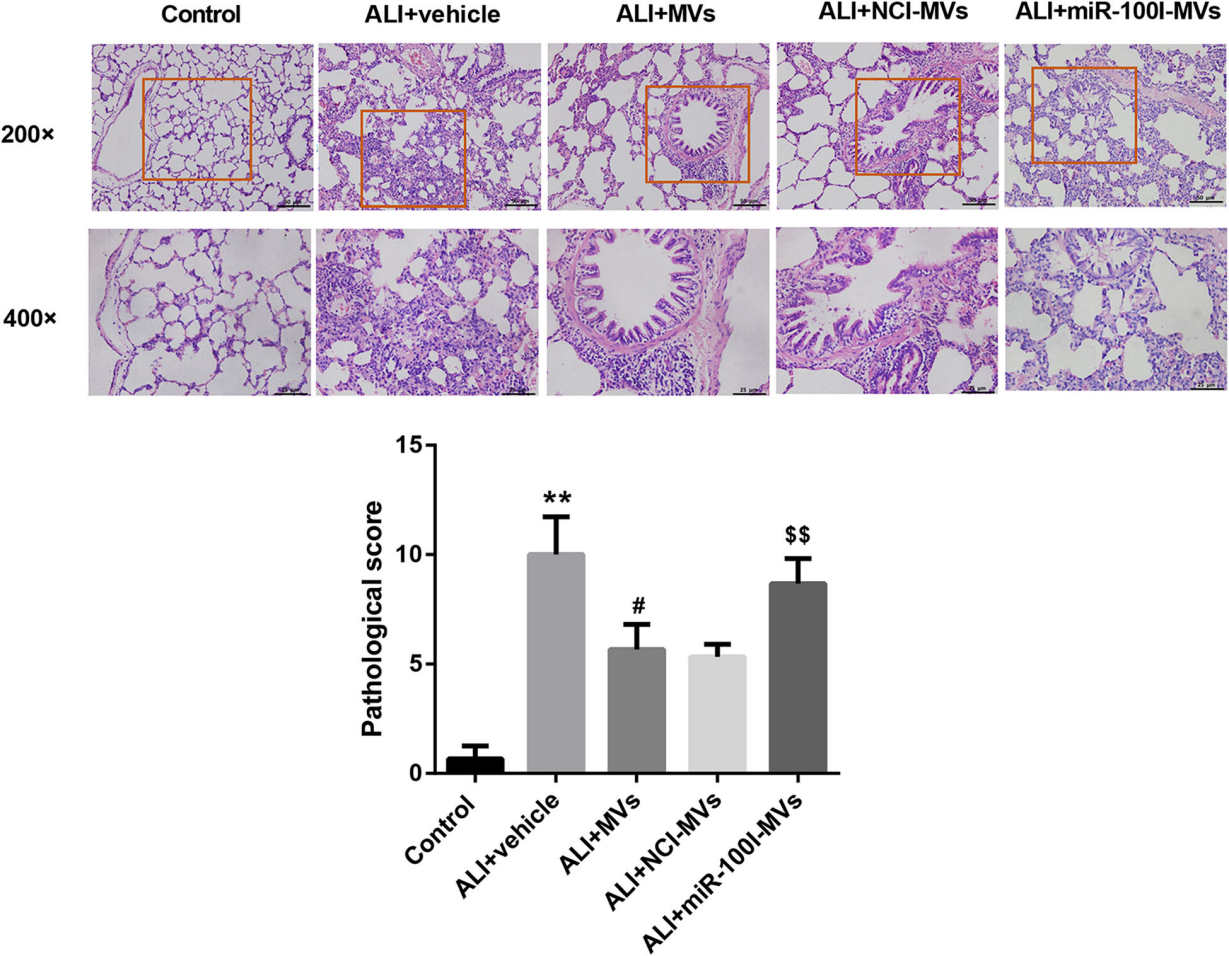
MSC-MVs ameliorate BLM-induced lung injury via transferring miR-100 in vivo*.* Representative histopathological changes of lung injury in ALI rats 1 week following administration with miR-100I-MVs. The upper and lower panels are magnification, × 200 and × 400, respectively. The injury was scored with a semi-quantitative grading system. **p < 0.01, vs. control; ^#^*p* < 0.01, vs. ALI+vehicle; ^$$^*p* < 0.01, vs. ALI+NCI-MVs

In addition, TUNEL staining demonstrated that the lung tissue cell apoptosis was remarkably promoted following BLM instillation, whereas was attenuated by MSC-MV administration for 48 h. However, compared with the NCI-MV group, the lung tissue cell apoptosis was enhanced in the miR-100I-MV group (Fig. 5g). Masson staining at 1-week post-injury showed that MSC-MVs could somewhat ameliorate the degree of lung injury induced by BLM administration. However, administration of miR-100I-MVs attenuated the anti-fibrosis effect of MSC-MVs on lung tissues (Fig. 6g). Furthermore, H&E staining at 1-week post-injury showed that miR-100I-MV administration impaired the MSC-MV-mediated alleviation of lung injury (Fig. 7). Together, these data indicate that MSC-MV administration effectively ameliorates BLM-induced ALI via miR-100 in rats.

## Discussion

To our knowledge, this study provided the first evidence revealing that miR-100 partly mediated the therapeutic effect of MSC-MVs on ALI in rats induced by BLM. The main findings of this study can be summarized as follows: (1) WJMSC-MV treatment attenuated BLM-induced apoptosis and inflammation in BLM-treated L2 cells and ameliorated BLM-induced lung apoptosis, inflammation, and fibrosis in BLM-induced ALI rats. (2) The beneficial effect of WJMSC-MVs was partly eliminated when miR-100 expression was knocked down in WJMSCs. (3) WJMSC-MV-transferred miR-100 mediated the therapeutic effect of WJMSC-MVs in ALI through enhancing autophagy by targeting mTOR.

The therapeutic potential of WJMSCs in BLM-induced lung injury has been highlighted previously [20]. However, the specific mechanism underlying the therapeutic benefit of MSCs for ALI is not fully illuminated. Likewise, their parent cells, MVs released from MSCs, were biologically active in ALI [21, 22]. Increasing evidence indicates that MSCs exert therapeutic effects in ALI partially by MSC-MVs [4, 5]. Convincing evidence has suggested that MSC-MVs ameliorated ALI by transferring various bioactive molecules with anti-apoptotic, anti-inflammatory, anti-oxidative, and anti-fibrotic properties to the injured tissue [5–7]. Zhu et al. [23] reported that MSC-MVs partly restored lung protein permeability and reduced inflammation in ALI mice induced by *E. coli* endotoxin, through transferring keratinocyte growth factor (KGF) mRNA to injured sites. Tang et al. [5] demonstrated that intratracheal administration of MSC-MVs ameliorated the lung inflammation and restored the pulmonary capillary permeability in a mouse model of LPS-induced ALI, partially through their content of Angiopoietin-1 (Ang-I) mRNA. Our group has recently demonstrated that MSC-MVs ameliorated BLM-induced ALI in rats partly via delivery of hepatocyte growth factor (HGF) [4]. These findings collectively suggested that KGF, Ang-I, or HGF mRNA might not be the exclusive way of contributing to the therapeutic benefit in ALI. In the current study, we confirmed the hypothesis that the therapeutic effects of MSC-MVs in ALI were also partly mediated by miR-100.

Convincing evidence has emphasized the tumor-suppressive role of miR-100 in lung cancer [24, 25]. Furthermore, a previous study has demonstrated that miR-100 inhibition by miR-100 inhibitor aggravated the LPS-induced cell injury and inhibited LPS-induced autophagy in LPS-treated WI-38 human lung fibroblasts, a cell model of pulmonary injury [15]. The regulation of autophagy by miR-100 has been examined by several studies [26, 27]. For example, Yu et al. reported that in osteosarcoma, miR-100 upregulation enhanced cell autophagy and apoptosis induced by cisplatin via targeted inhibiting of mTOR, which was known to be an important negative signal of autophagy [28]. More recently, miR-100-5p-abundant exosomes derived from infrapatellar fat pad MSCs have been shown to protect articular cartilage and ameliorate gait abnormalities by enhancing autophagy via targeted inhibition of mTOR in osteoarthritis [29]. In line with these findings, our results in this study showed that pretreatment with MHY1485, a mTOR activator that potently inhibits autophagy, effectively attenuated the anti-inflammatory and anti-apoptotic effect of MVs in BLM-treated L2 cells, suggesting that MSC-MVs attenuate the BLM-induced apoptosis and inflammation via elevating autophagy. Furthermore, we found that MSC-MV-transferred miR-100 restored the BLM-inhibited autophagy by targeting mTOR 3′-UTR. Therefore, we can clearly conclude that MSC-MV-transferred miR-100 mediated the anti-inflammatory and anti-apoptotic effect of MSC-MVs in BLM-stimulated L2 cells through enhancing autophagy by targeting and inhibiting mTOR. Changes of mTOR and autophagy level in lungs following miR-100I-MVs in BLM-induced ALI rats were consistent with results in BLM-treated L2 cells.

A pervious study showed that genetic knockdown of mTOR or activation of autophagy significantly attenuated, whereas inhibition of autophagy further augmented, LPS-induced inflammation response in human bronchial epithelial cells. Furthermore, mice with specific knockdown of mTOR in bronchial or alveolar epithelial cells exhibited significantly attenuated airway inflammation in response to LPS exposure [12]. These findings suggested that activation of autophagy was associated with the fall in inflammation, which was consistent with the results in the present study.

In this study, we aimed to study the mechanisms related to autophagy in this study. Considering the important role of mTOR/autophagy signaling in the progression of ALI, we selected miRNAs that can positively regulate autophagy. Coincidently, our TargetScan analysis predicted that mTOR was a putative target of miR-100, suggesting that miR-100 might positively regulate autophagy by inhibiting mTOR expression. Thus, miR-100 was selected in this study. Our results confirmed mTOR as a direct target of miR-100, which is one of the innovation points of this study. The results in this study also demonstrated that miR-100 indeed accounts for a part of the therapeutic effects of MSC-MVs in ALI. However, one of the limitations of this study is that we cannot rule out the possibility that other miRNAs also account for a part of the therapeutic effects of MSC-MVs, which requires further investigation.

In conclusion, our results in this study provided the first evidence revealing that WJMSC-MV-transferred miR-100 mediated, at least in part, the therapeutic effect of WJMSC-MVs in ALI through restoring mTOR signal-mediated inhibition of autophagy.

## Data Availability

The datasets used and/or analyzed during the current study are available from the corresponding author on reasonable request.

## Abbreviations

3′-UTR: 3′-Untranslational region
ALI: Acute lung injury
Ang-I: Angiopoietin-1
ANOVA: Analysis of variance
BAL: Bronchoalveolar lavage
BALF: Bronchoalveolar lavage fluid
BLM: Bleomycin
CVF: Collagen volume fraction
ECL: Enhanced chemiluminescence
ELISA: Enzyme-linked immunosorbent assay
FBS: Fetal bovine serum
FCM: Flow cytometry
FITC: Fluorescein isothiocyanate
HGF: Hepatocyte growth factor
IFN-γ: Interferon-γ
IL-6: Interleukin-6
IL-8: Interleukin-8
KGF: Keratinocyte growth factor
LPS: Lipopolysaccharide
MSCs: Mesenchymal stem cells
MTOR: Mechanistic target of rapamycin
Mut: Mutant
MVs: Microvesicles
PI: Propidium iodide
QRT-PCR: Quantitative real-time PCR
SD: Standard deviation
SPF: Specific pathogen-free
TEM: Transmission electron microscope
TNF-α: Tumor necrosis factor-α
TUNEL: TdT-mediated dUTP nick-end labeling
WT: Wild type
